# Genomic characterization of Streptococcus pneumoniae isolates obtained from carriage and disease among paediatric patients in Addis Ababa, Ethiopia

**DOI:** 10.1099/mgen.0.001376

**Published:** 2025-03-18

**Authors:** Abel Abera Negash, Ana Ferreira, Daniel Asrat, Abraham Aseffa, Piet Cools, Leen Van Simaey, Mario Vaneechoutte, Stephen D. Bentley, Stephanie W. Lo

**Affiliations:** 1 Armauer Hansen Research Institute (AHRI), Addis Ababa, Ethiopia; 2 Parasites and Microbes, Wellcome Sanger Institute, Hinxton, UK; 3 Department of Microbiology, Immunology and Parasitology, School of Medicine, Addis Ababa University, Addis Ababa, Ethiopia; 4 Laboratory Bacteriology Research, Department of Diagnostic Sciences, Faculty of Medicine and Health Sciences, Ghent University, Ghent, Belgium; 5 Milner Centre for Evolution, Department of Life Sciences, University of Bath, Bath, UK; 6 The Great Ormond Street Institute of Child Health, University College London, London, UK

**Keywords:** Ethiopia, GPSC1, GPSC10, PCV10, serotype 19A, *Streptococcus pneumoniae*

## Abstract

**Background and aims.** Despite the introduction of pneumococcal conjugate vaccines (PCVs), *Streptococcus pneumoniae* still remains an important cause of morbidity and mortality, especially among children under 5 years in sub-Saharan Africa. We sought to determine the distribution of serotypes, lineages and antimicrobial resistance of *S. pneumoniae* from carriage and disease among children presenting to health facilities, 5–6 years after the introduction of PCV10 in Ethiopia.

**Methods.** Whole-genome sequencing (WGS) was performed on 103 *S. pneumoniae* (86 from nasopharyngeal swabs, 4 from blood and 13 from middle ear discharge) isolated from children aged <15 years at 3 healthcare facilities in Addis Ababa, Ethiopia, from September 2016 to August 2017. Using the WGS data, serotypes were predicted, isolates were assigned to clonal complexes, global pneumococcal sequence clusters (GPSCs) were inferred and screening for alleles and mutations that confer resistance to antibiotics was performed using multiple bioinformatic pipelines.

**Results.** The 103 *S*. *pneumoniae* isolates were assigned to 38 serotypes (including nontypeable) and 46 different GPSCs. The most common serotype was serotype 19A. Common GPSCs were GPSC1 [14.6% (15/103), sequence type (ST) 320, serotype 19A], GPSC268 [8.7% (9/103), ST 6882 and novel STs; serotypes 16F, 11A and 35A] and GPSC10 [8.7% (9/103), STs 2013, 230 and 8804; serotype 19A]. The four invasive isolates were serotype 19A (*n*=2) and serotype 33C (*n*=2). Resistance to penicillin (>0.06 µg ml^−1^, CLSI meningitis cutoff) was predicted in 57% (59/103) of the isolates, and 43% (25/58) penicillin-binding protein allele combinations were predicted to be associated with penicillin resistance. Resistance mutations in *folA* (*I100L*) and/or *folP* (indel between fifty-sixth and sixty-seventh aa) were identified among 66% (68/103) of the isolates, whilst tetracycline (*tetM*) and macrolide (*ermB* and *mefA*) resistance genes were found in 46.6% (48/103), 20.4% (21/103) and 20.4% (21/103) of the isolates, respectively. Multidrug resistance (MDR) (≥3 antibiotic classes) was observed in 31.1% (32/103) of the isolates. GPSC1 and GPSC10 accounted for 46.8% (15/32) and 18.7% (6/32) of the overall MDR.

**Conclusion.** Five to 6 years after the introduction of PCV10 in Ethiopia, the *S. pneumoniae* obtained from carriage and disease among paediatric patients showed diverse serotype and pneumococcal lineages. The most common serotype identified was 19A, expressed by the MDR lineages GPSC1 and GPSC10, which is not covered by PCV10 but is included in PCV13. Continued assessment of the impact of PCV on the population structure of *S. pneumoniae* in Ethiopia is warranted during and after PCV13 introduction.

**Impact Statement**

This study provides a detailed analysis of the genomic features of carriage and disease *Streptococcus pneumoniae* isolates from paediatric patients in Addis Ababa, Ethiopia, collected 5–6 years after the introduction of PCV10 in the country and will serve as the baseline to evaluate the impact of PCV13 that was introduced in July 2020. It revealed serotype 19A as the most common serotype. This finding supports the introduction of PCV13, which includes serotype 19A in its formulation to provide protection against it. We also identified high-risk pneumococcal lineages (GPSC5, 10 and 268) that potentially mediate serotype replacement after PCV13 introduction. The high prevalence of genome-predicted penicillin resistance at the CLSI meningitis cutoff suggests that alternative antibiotic options should be considered in meningitis cases. Our study highlighted the importance to continue performing genomic surveillance of carriage and disease pneumococcal isolates in Ethiopia to assess the impact of the recently introduced PCV13 on the serotype distribution and population structure of *S. pneumoniae*.

## Data Summary

Genome sequences are deposited at ENA with accession numbers ERR9796440-ERR9990857 and ERR10419695-ERR10419739, and a phylogenetic snapshot is available at https://microreact.org/project/gps2-ethiopia-nov24. The metadata, *in silico* typing output, *de novo* assemblies and annotation files are available at GPS Monocle database (https://data-viewer.monocle.sanger.ac. uk/project/gps). The authors confirm that all supporting data have been provided within the article, the GPS Monocle database or through supplementary data files.

## Introduction

*Streptococcus pneumoniae* is an important cause of morbidity and mortality [1]. It caused an estimated 318 000 deaths in 2015 in children aged 1–59 months globally, and more than 50% of these deaths were in the African continent. This is despite the introduction and use of pneumococcal conjugate vaccines (PCVs) between the years 2000 and 2015, which already caused a 50% reduction in pneumococcal deaths [2].

Currently, 107 pneumococcal serotypes have been identified [3], whilst the available conjugate vaccines target only a limited number of the most prevalent serotypes causing invasive disease [4]. The introduction of PCV in the routine immunization of infants has proved to be effective in reducing vaccine type (VT) carriage by up to 82% [5,7] and the burden of pneumococcal diseases caused by VT pneumococci by up to 100% [8,10]. In addition, the indirect benefit of PCV against VT colonization and disease in the unvaccinated population (herd protection) has also been documented [11]. The overall pneumococcal carriage prevalence has however stayed the same mainly due to a significant increase in non-vaccine type (NVT) pneumococci carriage, indicating that the NVTs are replacing VT pneumococci [12]. Likewise, an increase in pneumococcal disease due to NVT was also observed in many European countries and the USA after the introduction of PCV7 [13]. A similar trend has been reported in both European and African countries after the introduction of PCV10 and PCV13 [14,15].

Whole-genome sequencing (WGS) has become a valuable tool for understanding the distribution of pneumococcal lineages, population evolution and serotype switching and replacement [16,17]. The international definition of pneumococcal population structure known as global pneumococcal sequence clusters (GPSCs) was defined to provide further context on the distribution of serotypes, antibiotic resistance and invasiveness across pneumococcal lineages for epidemiological analysis of pneumococcal populations in the post-PCV era [17].

Since PCVs target only a limited number of pneumococcal serotypes, their widespread use has increased both the nasopharyngeal carriage and invasive pneumococcal diseases (IPDs) due to non-vaccine serotypes, and serotype 19A is one of the predominant non-vaccine serotypes that emerged after the introduction of PCV7 in 2000 [18]. In addition, compared with other non-vaccine serotypes, serotype 19A exhibited higher invasive disease potential [19]. An increase in serotype 19A carriage and IPD and associated high level of multidrug resistance (MDR) has also been reported from countries that introduced PCV10 [20,21]. One of the main factors that is attributed to the emergence of serotype 19A in the post-PCV7 and PCV10 era is inter-strain genetic recombination, which results in the acquisition of new capsular serotype, also known as capsular switching [22]. In a recent study, using WGS, Corcoran *et al*. were able to identify sub-clade/variants of serotype 19A associated with IPD in vaccinated children in Ireland, indicating vaccine failures after PCV13 introduction [23] and highlighting the importance of WGS in studying the molecular epidemiology of *S. pneumoniae*.

In Ethiopia, the PCV10 (serotypes 1, 4, 5, 6B, 7F, 9V, 14, 18C, 19F and 23F) was introduced in October 2011 as a three-dose primary series without a booster dose (3p+0) [24]. This was replaced with PCV13 (PCV10 serotypes plus serotypes 3, 6A and 19A) in July 2020 [25] and is currently the only PCV used. The transition from PCV10 to PCV13 was partly based on the evidence that after the introduction of PCV10 in Ethiopia, pneumococcal serotype 19A became predominant in both carriage [26] and disease [27].

The aim of the study was to determine the distribution of serotypes, lineages and antibiotic resistance among carriage and disease *S. pneumoniae* isolates collected from paediatric patients in Addis Ababa, Ethiopia, 5–6 years after the introduction of PCV10.

## Methods

### Study design

**Fig. 1.**
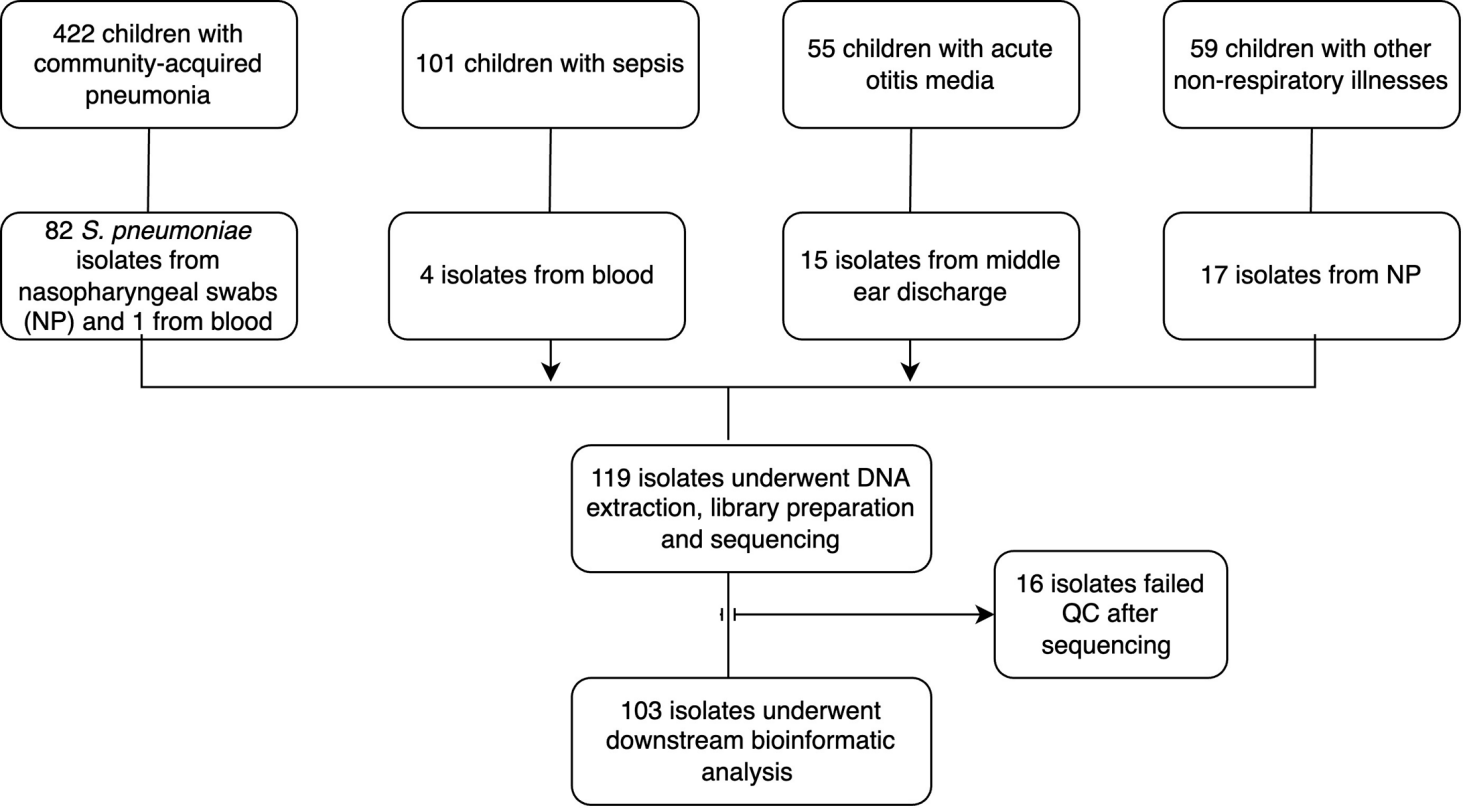
Flowchart depicting the number of children recruited, number of *S. pneumoniae* isolates identified and those that were characterized in this study.

This study is a retrospective analysis of *S. pneumoniae* isolates that were collected as part of prospective studies performed from September 2016 to August 2017 in Addis Ababa, Ethiopia, among children with pneumonia (422), sepsis (101) and acute otitis media (AOM) (55) and children with non-respiratory illnesses besides sepsis and AOM (59) (Fig. 1).

### Bacterial isolates and serotyping

The *S. pneumoniae* isolates included in the present study were isolated from children aged 0–15 years attending paediatric emergency departments in four major healthcare facilities in Addis Ababa, Ethiopia (Tikur Anbessa Hospital, Yekatit 12 Hospital, Girum Hospital and Dr. Yared Pediatric Specialty Center) from September 2016 to August 2017. During the study period, 119 pneumococcal isolates were collected, and their serotype distribution has been previously described [26,29]. The 119 pneumococcal isolates were subject to WGS, and 87% (103/119) of the samples passed quality control. The 103 pneumococcal isolates were included in all subsequent analyses. They were *S. pneumoniae* isolated from nasopharyngeal swabs of children with community-acquired pneumonia (*n*=73), nasopharyngeal swabs of children with non-respiratory illnesses such as gastroenteritis and congenital heart disease (*n*=13), blood of children with pneumonia (*n*=1) or sepsis (*n*=3) and middle ear discharge of children with AOM (*n*=13) and their serotypes, which were determined by Quellung reaction [30] using antisera, obtained from the Statens Serum Institut (Copenhagen, Denmark).

### Genomic characterization

Genomic DNA was extracted using PureLink™ Genomic DNA Kit (Invitrogen, Carlsbad, CA). The Illumina NovaSeq platform at the Wellcome Sanger Institute sequencing facility (Cambridge, UK) was used to produce paired-end reads with a length of ~150 bp. Reads were trimmed, adapters were removed or speciation was done using Wellcome Sanger Institute’s internal pipeline. Assembly of sequences was performed using the velvet version 1.2.10 with Velvet Optimiser version 2.26 [31], and sequences were mapped to the genome sequence PMEN1 of strain ATCC 700669 (NCBI accession number FM211187). Quality score parameters used were as follows: sequencing depth of >20×, >60% map to reference genome PMEN1, assembly length of 1.9–2.3 Mb, less than 500 contigs and less than 220 heterozygous SNP sites. The assembled reads were annotated using Prokka version 1.14.5 (https:// github.com/tseemann/prokka) [32]. Serotypes were inferred from genome data using SeroBA version 1.0.7 (https://github.com/ sanger-pathogens/seroba) [33]. WGS-based multi-locus sequence typing was performed using MLST version 2.22.0 [34], and sequence types (STs) were assigned clonal complex (CC) using a single-locus variant (SLV) threshold [17], and clustering the sequences into lineages and GPSCs was inferred using PopPUNK version 2.6.3 (https://poppunk.net/) with GPSC reference database version 6 [35]. We inferred resistance for 15 antibiotics, including penicillin (PEN), amoxicillin (AMO), meropenem (MER), cefotaxime (TAX), ceftriaxone (CFT), cefuroxime (CFX), erythromycin (ERY), clindamycin (CLI), linezolid (LZO), cotrimoxazole (COT), tetracycline (TET), levofloxacin (LFX), chloramphenicol (CHL), rifampin (RIF) and vancomycin (VAN) from the genomic data using the CDC beta-lactam resistance pipeline for the pneumococcus [17,36, 37]. We used the Clinical and Laboratory Standards Institute (CLSI) (https://clsi.org/standards/) break points for interpretation of resistance. MDR was defined as resistance to three or more classes of antibiotics. Phylogenetic analysis was performed by constructing a maximum- likelihood tree using FastTree version 2.1.10 [38] with General Time Reversible substitution model based on SNPs extracted from an alignment generated by mapping reads to the reference genome of *S. pneumoniae* ATCC 700669 (NCBI accession number FM211187) using Smalt (version 0.7.4; https://github.com/rcallahan/smalt). To place a global context in the common pneumococcal lineage, we downloaded the genomes belonging to the lineage of interest from the GPS Monocle database (https://data-viewer.monocle.sanger.ac.uk/project/gps, last accessed on 9 October 2024). The GPS genomes, together with the genomes in this study, were subject to build a recombination-free phylogenies using GUBBINS (version 3.2.1.) based on SNPs extracted from an alignment generated by mapping reads to the respective GPSC-specific reference genome using Snippy (version 2.6.0). The reference genomes for GPSC1 and GPSC10 are under the accession number of CP000921 and ERS740618, respectively.

### Ethical considerations

The study procedures were in accordance with the Helsinki Declaration. The study protocol was approved by the AHRI/All Africa Leprosy Rehabilitation and Training Hospital (ALERT) Ethical Review Committee (AAERC) (PO/017/15) and the National Research Ethics Review Committee (No. 310/194/17). An official permission letter was obtained from all the study sites. A written informed assent and consent was obtained from study participants and parents or guardians of children, respectively, before including them in the study. The study participant’s right to refuse or not give samples without affecting their routine medical services was granted. Samples were coded to keep the confidentiality of the study participants’ personal information.

## Results

### Prevalence of pneumococcal serotypes

Among the 119 isolates sequenced, 16 failed quality control and downstream bioinformatic analysis was therefore performed on the remaining 103 isolates (Table S1, available in the online Supplementary Material). Genomic inference of serotype was reliable with 75% (77/103) to the serotype level and 85% (88/103) to the serogroup level compared with serotype determined by Quellung reaction (Table S2). The 15 pneumococcal isolates with discordant phenotypic and *in silico* serotype did not belong to a particular GPSC/ST, and no systematic mistyping was observed. The *in silico* typing provided unequivocal typing results by recognizing the serotype-specific genes and mutations; therefore, *in silico* serotypes were used in the subsequent analyses. In this collection, we identified 38 different serotypes, including 1 isolate that was untypable by SeroBA and was non-capsulated by Quellung reaction. Based on SeroBA, the most common serotype was serotype 19A (28.4%, 29/103), followed by 16F (6.8%, 7/103). Serotype 19A was the most common serotype in all three sample types (nasopharyngeal swabs, blood and middle ear discharge). Among the serotypes identified, only 7.8% (8/103) were PCV10 (GSK) serotypes [i.e. serotypes 6B (*n*=1), 7F (*n*=1), 9V (*n*=1), 14 (*n*=1), 19F (*n*=3) and 18C (*n*=1)]. PCV13 (Pfizer) would increase the coverage to 38.8% (40/103). The overall coverage for PCV10 (SII), PCV15 (Merck), PCV20 (Pfizer), PCV24 (Merck) and IVT-25 (Inventprise) were 37.9% (39/103), 38.8% (40/103), 48.5% (50/103), 51.5% (53/103) and 56.3% (58/103), respectively.

### Pneumococcal lineages

**Fig. 2.**
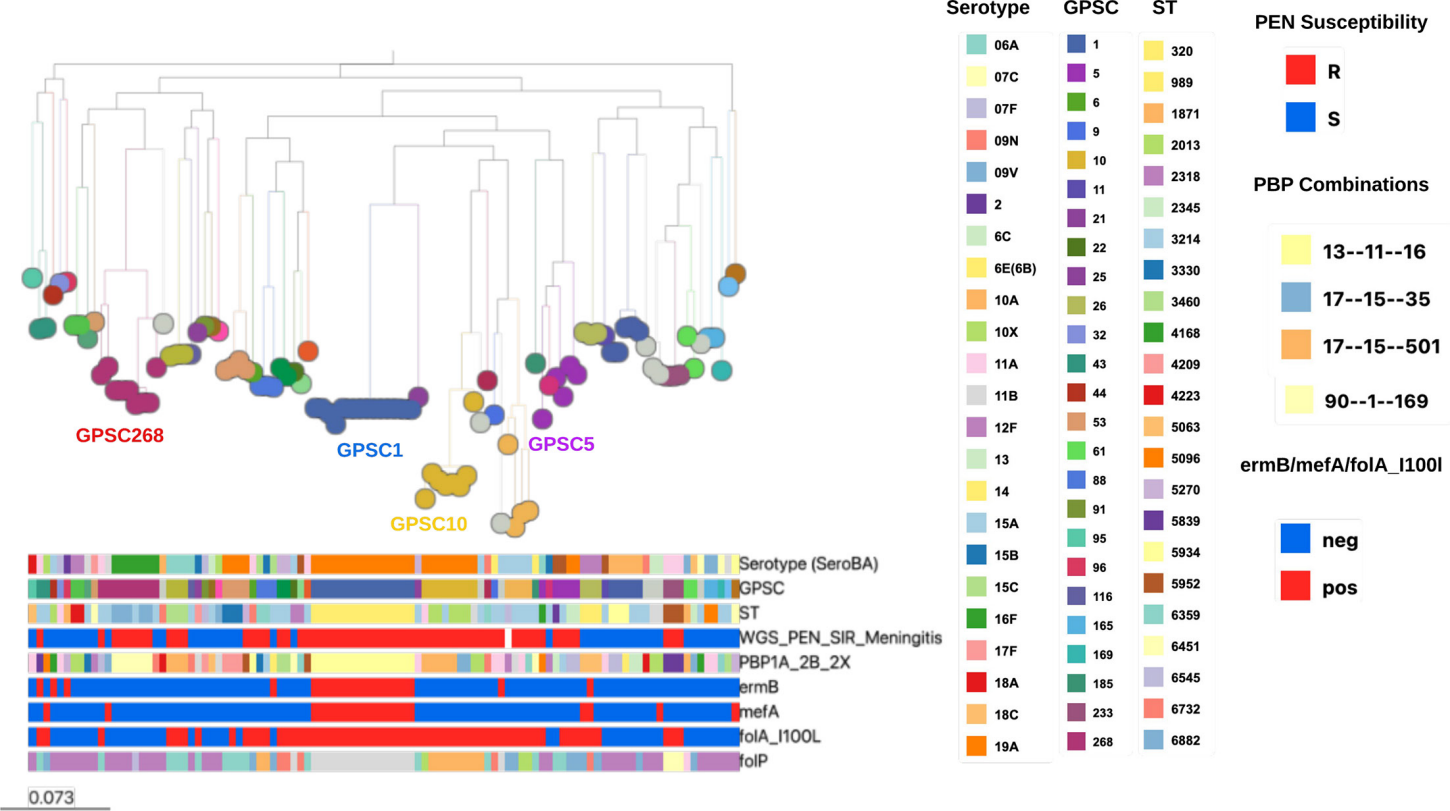
Phylogenetic map indicating the serotypes, lineages (GPSCs) and STs, penicillin resistance and resistance genes for 103 *S. pneumoniae* isolates from paediatric patients in Addis Ababa, Ethiopia. The maximum-likelihood tree was constructed with FastTree using a generalized time reversible model. The most common serotypes, GPSCs, STs and resistance genes are depicted in the legend. PBP, penicillin-binding protein; PEN, penicillin; erm, erythromycin ribosome methylase; mef, macrolide efflux; fol, dihydrofolate reductase.

The 103 *S*. *pneumoniae* isolates were assigned to 46 different GPSCs (Fig. 2 and Table S3), of which 40 were already present in the GPSC reference database (https://www.pneumogen.net/gps/#/training#command-line). Six novel GPSCs were GPSC1004 (*n=1), 1005 (*n*=2), 1013 (*n*=1), 1014 (*n*=1), 1015 (*n*=1) and 1016 (*n*=1). The most common GPSCs were GPSC1 [ST 320, serotype 19A; 14.6% (15/103)], GPSC268 [ST 6882 (*n*=5), SLV (*n*=1), double-locus variant (DLV) (*n*=1) and triple-locus variant (*n*=2); serotypes 16F, 11A and 35A; 8.7% (9/103)] and GPSC10 [STs 2013 (*n*=5), 230 (*n*=1), 8804 (*n*=1) and 2013-SLV (*n*=1); serotype 19A; 7.8% (8/103)] (Table 1). GPSC1 was the most common lineage in isolates from both nasopharyngeal swabs (8/87) and middle ear discharge (6/13). The 103 isolates sequenced were assigned into 36 known STs (*n*=73) and 28 novel STs (*n*=30). Among known STs, the most common was ST320. The most common serotype 19A was expressed by three lineages (GPSC1, GPSC10 and GPSC5), and all the serotype 16F belonged to GPSC268.

**Table 1.** Top five GPSCs, associated serotypes and sample sources among paediatric patients in Addis Ababa, Ethiopia

GPSC		**ST**		Associated serotype	Sample type	*N*
**Type**	***N* (%**)	**Type**	***N* (%**)			
1	15 (14.6)	320	15 (14.4)	19A	Blood	1
					MED	6
					NP	8
268	9 (8.7)	6882	5 (4.85)	16F	MED	1
					NP	4
		ST6882-SLV and TLV	2		NP	2
		ST6882-DLV	1	11A	NP	1
		ST6882-TLV	1	35C	NP	1
10	8 (7.8)	2013	5 (4.9)	19A	Blood	1
					MED	1
					NP	3
		230	1 (0.97)		NP	1
		8804	1 (0.97)		MED	1
		ST2013-SLV	1 (0.97)		NP	1
5	5 (4.9)	2345	2 (1.94)	19A	MED	1
					NP	1
		5839	1 (0.97)	21	NP	2
		5839-SLV	1 (0.97)		NP	1
		4168		23A	NP	1
584	5 (4.9)	5934	3 (2.91)	34	NP	3
		5934-SLV	2 (1.94)		NP	2

MED, middle ear discharge; NP, nasopharyngeal swab; TLV, triple-locus variant.

**Fig. 3.**
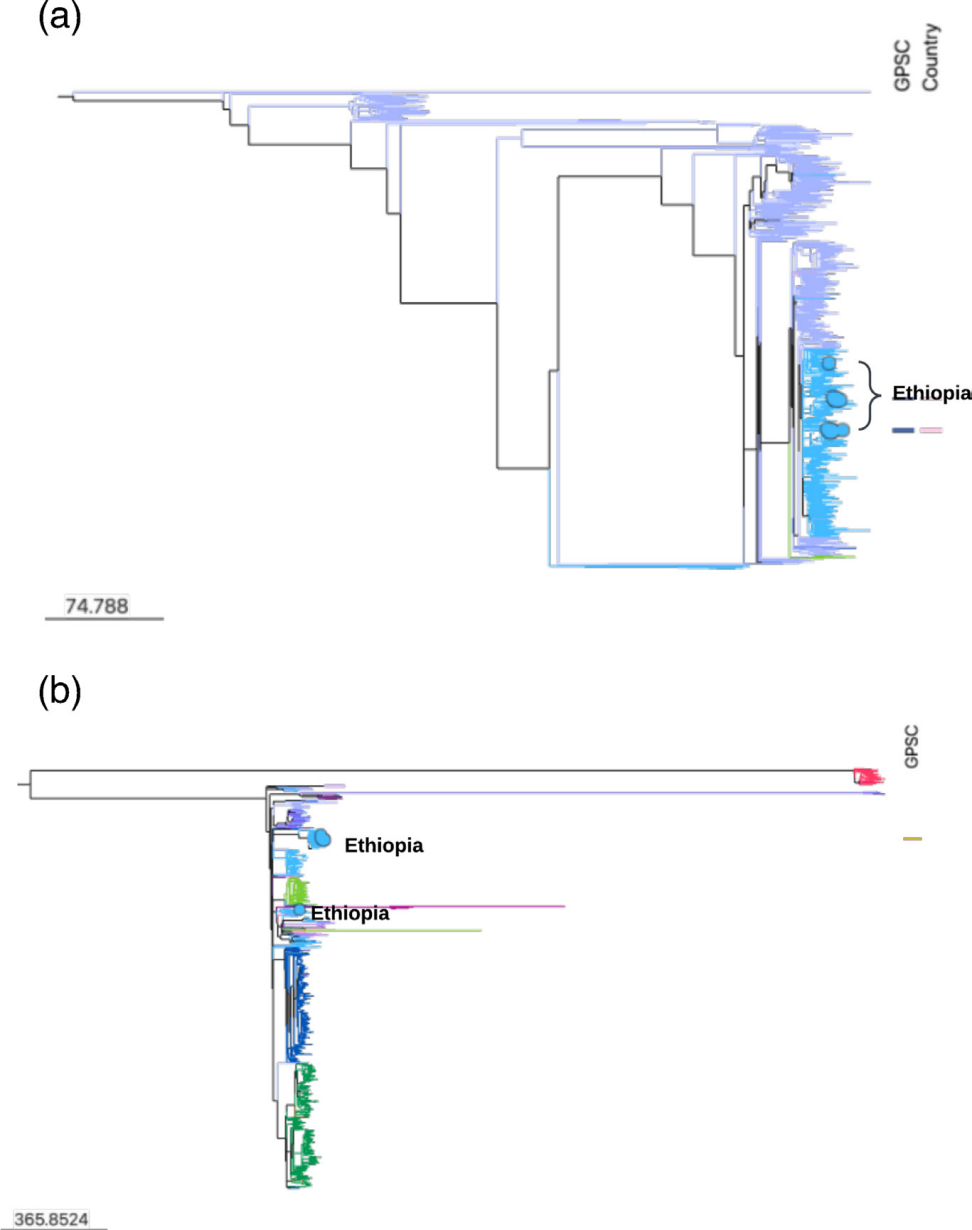
Global phylogeny of GPSC1 serotype 19A (**a**) and GPSC10 serotype 19A (**b**). The recombination-free phylogeny was constructed using GUBBINS version 3.2.1. The blue encircled balloons indicate *S. pneumoniae* serotype 19A lineages from Ethiopia. Links to access the phylogenetic trees are https://microreact.org/project/gps2-gpsc1-ethiopiapaper and https://microreact.org/project/gps1-gpsc10-ethiopiapaper.

To detect the potential clonal expansion of GPSC1 and GPSC10 in Ethiopia, we created a global phylogeny of these two pneumococcal lineages with the GPS dataset and collection in this study. The Ethiopian GPSC1 19A isolates were all clustered in the 19A sub-lineage (Fig. 3a). Within the 19A sub-lineage, Ethiopian isolates were sporadically clustered with isolates from other geographical origins, indicating that the clonal expansion of GPSC1 was not detected and multiple importations of GPSC1 into Ethiopia were possible.

In contrast, a cluster of Ethiopian GPSC10 isolates (*n*=6) was detected, whilst two isolates clustered elsewhere, suggesting that there could be a combination of clonal expansion of GPSC10-19A in Ethiopia over time and multiple importations of GPSC10 into Ethiopia (Fig. 3b).

### *In silico* predicted phenotypic antimicrobial susceptibility pattern and resistance genes

**Fig. 4.**
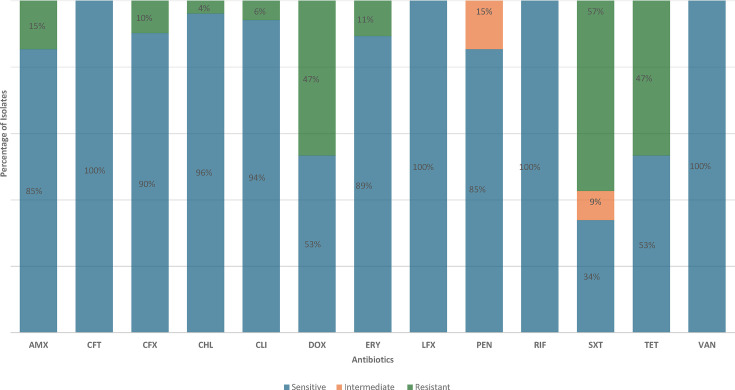
*In silico* predicted antimicrobial susceptibility pattern of *S. pneumoniae* isolates among paediatric patients in Addis Ababa, Ethiopia. AMX, amoxicillin; CFT, cefotaxime; CFX, cefuroxime; CHL, chloramphenicol; CLI, clindamycin; DOX, doxycycline; ERY, erythromycin; LFX, levofloxacin; PEN, penicillin; RIF, rifampin; SXT, trimethoprim-sulfamethoxazole; TET, tetracycline; VAN, vancomycin; Non-M, non-meningitis; M, meningitis.

For all isolates, phenotypic antimicrobial susceptibility was previously performed using a combination of Kirby–Bauer disc diffusion and minimum inhibitory concentrations (MICs) using Etest strips (bioMérieux, Marcyl’Étoile, France) for penicillin and erythromycin [28]. In this study, the antimicrobial susceptibility pattern was inferred from genome data (summarized in Fig. 4). Comparison of the phenotypic and *in silico* predicted antimicrobial susceptibility indicated that there was a concordance of 96.1% (99/103) for penicillin using meningitis break points and 86.4% (89/103) using non-meningitis break points. Using meningitis break points, phenotypic penicillin resistance was 59.2% (61/103) compared with the *in silico* predicted resistance of 57% (57/103). Similarly, there was 86.4% (89/103) concordance for erythromycin. Phenotypic erythromycin resistance was 33% (34/103) compared with *in silico* predicted resistance of 25.2 (26/103). Isolates with discordant antimicrobial susceptibility pattern have been summarized in Table S4.

Overall, based on the results of the *in silico* predicted antimicrobial sensitivity testing, 74% (76/103) of pneumococcal isolates were resistant to at least 1 antibiotic. Using a CLSI meningitis cutoff, >50% of the isolates in three sample sources were resistant to penicillin (Table 2). Based on the CLSI non-meningitis cutoff, 15 pneumococcal isolates [blood (*n*=1), middle ear swab (*n*=6) and nasopharyngeal swabs (*n*=8)] were categorized as intermediately resistant to penicillin (predicted MIC value=4 µg ml^−1^) and to cefotaxime (predicted MIC value=2 µg ml^−1^), whilst others were categorized as susceptible. All the intermediate non-susceptible penicillin isolates were serotype 19A. Using meningitis breakpoints, penicillin resistance was predicted in 57% (59/103) of the isolates. MDR (≥3 antibiotic classes) was observed in 31.1% (32/103) of the isolates. GPSC1 and GPSC10 accounted for 46.8% (15/32) and 18.7% (6/32) of the overall MDR. Almost half, 46.8% (15/32), of MDR isolates were serotype 19A, expressing GPSC1 lineage (ST 320). Among the four invasive isolates from children with pneumonia (*n*=1) and sepsis (*n*=3), two were serotype 19A (GPSC1, ST 320 and GPSC10, 2013) and were MDR, whilst the other two were serotype 33C (ST 5029).

**Table 2.** Prevalence of antimicrobial resistance in 103 pneumococcal isolates by sample source

Antibiotic	Sample source, *n* (%)			Total
	Blood (*n*=4)	Middle ear discharge (*n*=12)	Nasopharyngeal swab (*n*=87)	
Penicillin*	2 (50)	12 (100)	45 (52)	59 (57)
Amoxicillin	1 (25)	6 (50)	8 (9)	15 (15)
Cefuroxime	1 (25)	8 (67)	16 (18)	25 (24)
Cefotaxime	1 (25)	6 (50)	8 (9)	15 (15)
Meropenem	1 (25)	6 (50)	8 (9)	15 (15)
Chloramphenicol	0	0	4 (5)	4 (4)
Clindamycin	1 (25)	6 (50)	14 (16)	21 (20)
Erythromycin	1 (25)	6 (50)	19 (22)	26 (25)
Cotrimoxazole	2 (50)	11 (92)	46 (53)	59 (57)
Tetracycline	2 (50)	9 (75)	37 (43)	48 (47)

* The predicted MIC value was interpreted based on the CLSI guideline meningitis cutoff. Penicillin and cefotaxime MIC values are >0.06 and >0.5 µg ml−1 and are considered resistant, respectively.

**Fig. 5.**
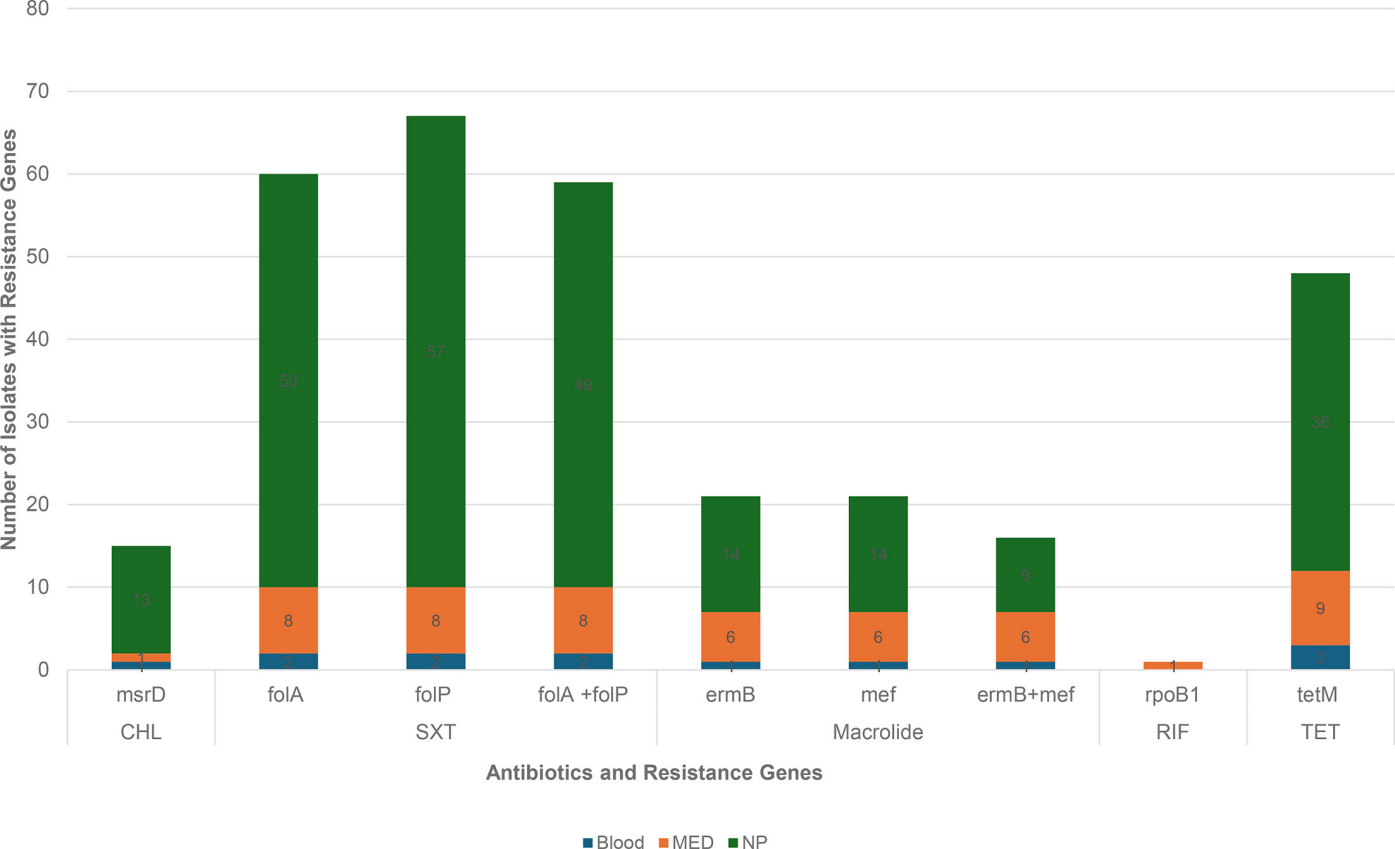
Distribution of non-beta lactam antimicrobial resistance genes among *S. pneumoniae* isolates from paediatric patients in Addis Ababa, Ethiopia. CHL, chloramphenicol; SXT, trimethoprim-sulfamethoxazole; RIF, rifampin; TET, tetracycline; MED, middle ear discharge; NP, nasopharyngeal swab.

Among isolates predicted to be resistant to penicillin (57) using CLSI meningitis break points, the most frequent penicillin- binding protein (PBP) combination was 13-11-16, 22.8% (13/57), followed by 17-15-501, 12.2% (7/57), and 90-1-169, 10.5% (6/57) (Fig. 2). All the resistant isolates with PBP combination of 13-11-16 are MDR GPSC1, serotype 19A, ST320. Overall, 43.8% (25/57) of PBP allele combinations were predicted to be associated with penicillin resistance. Resistance mutations in *folA* (*I100L*) or *folP* (indel between fifty-sixth and sixty-seventh aa) were identified among 66% (68/103) of the isolates, whilst tetracycline (*tetM*) and macrolide (*ermB* and *mefA*) resistance genes were found in 46.6% (48/103), 20.4% (21/103) and 20.4% (21/103) of the isolates, respectively (Fig. 5). MDR was predicted in 56.3% of the isolates, and the most common MDR lineage was GPSC1 (ST 320, serotype 19A).

## Discussion

In this study, we analysed the genomic characteristics of 103 *S. pneumoniae* isolates from children with pneumonia (73 isolates from nasopharyngeal swabs and 1 isolate from blood), non-respiratory illnesses (13 isolates from nasopharyngeal swabs), sepsis (3 isolates from blood) and AOM (13 isolates from middle ear discharge) in Addis Ababa, Ethiopia, 5–6 years after the introduction of PCV10 in the country. The predominant serotypes were non-PCV10 serotypes 19A (28.4%) mainly expressed by GPSC1 (CC320) and GPSC10 (CC230), followed by 16F (7.8%) expressed by only GPSC268 (CC6882). Serotype 19A is included in currently available PCV13 and higher valency PCVs such as PCV15 and PCV20. Serotype 16F is not included in any PCVs that are currently licensed for children’s use. It is included in PCV21 (CAPVAXIVE, Merck), PCV25 (IVT-25 Inventprise) and PCV31 (VAX-31, Vaxcyte). CAPVAXIVE is designed for individuals aged≥18 years old and is now FDA approved. The latter two are still under development.

In Mozambique, a 40% increase in the colonization prevalence for the three PCV13-specific serotypes (3, 6A and 19A) was reported 3 years after introducing PCV10 [39]. In Nepal and Pakistan, a significant increase in the prevalence of serotype 19A has been reported 4 and 5 years after the introduction of PCV10, respectively [40,41]. In Belgium, 4 years after a shift from PCV13 to PCV10, the prevalence of serotype 19A carriage among children in day care centres doubled and became the most dominant serotype [42]. A similar trend was also observed in the disease-causing pneumococcal population in Belgium; a rapid reemergence of serotype 19A driven by ST416 (a DLV of ST199 and belonged to GPSC4) and ST994 belonging to GPSC146 was observed after switching from PCV13 to PCV10 [43]. Comparable results were reported in a study from Slovakia, where serotype 19A was the predominant serotype causing AOM among children after the introduction of PCV10 [44]. In Brazil, which introduced PCV10 in 2010, serotype 19A was the leading serotype in all ages from 2014 to 2021 and was responsible for 28.2–44.6% of all IPD cases in children under 5 years [45]. Since pre-PCV10 serotype data in Ethiopia are scarce, and the association between the prevalence of serotype 19A and the introduction of PCV10 cannot be determined, it will be important to continue to study the epidemiology of serotype 19A and the impact of PCV13, which has replaced PCV10 in the immunization schedule.

The most common pneumococcal lineage in this study was GPSC1 (CC320), and it accounted for 51% of the most common serotype 19A that are not covered by the PCV10 but PCV13. In Brazil, in the post-PCV10 era, GPSC1 was the third most common lineage causing IPD across all ages and the most common one among children aged<5 years [46]. Genomic characterization of *S. pneumoniae* isolates from cerebrospinal fluid, nasopharyngeal swabs and non-sterile site swabs in Russia also indicated that GPSC1 was the most common lineage [47]. In a study that investigated pneumococcal carriage dynamics before and after antibiotic treatment in 965 unvaccinated infants and a subset of their mothers in Thailand, GPSC1 was the most common lineage detected after treatment [48].

Globally, GPSC1 has mediated serotype replacement in disease-causing and carriage pneumococcal populations since the introduction of PCV7 in multiple countries [46,49, 50]. Majority of GPSC1 expressed serotypes 19F and 19A, to a lesser extent of other serotypes such as serotypes 3, 6C, 14 and 23F (https://microreact.org/project/gpsGPSC1). Globally, the genetic background of GPSC1 had a higher invasive disease potential independent of serotype and was consistently found to be MDR [17].

Interrogation of PubMLST and GPS Monocle databases (last accessed on 18 October 2024), GPSC268 (CC6882) was mainly found in the African continent, except for one blood culture pneumococcal isolate recovered in Norway from a 45-year-old individual in 2015. Apart from Ethiopia, GPSC268 was detected in Malawi (*n*=7, four from blood culture and three from nasopharyngeal swabs of healthy individuals) and Kenya (*n*=2, nasopharyngeal swabs from healthy individuals) in Africa. Majority of them expressed serotype 16F, except for four Ethiopian samples expressing serotypes 11A (*n*=2), 23F (*n*=1) and 35C (*n*=1). Of these four GPSC268 isolates, two were carriage samples (23F and 11A) from Global Strain Bank at US CDC with an unknown collection of the year whilst the other two were in this study.

GPSC10 is the third common pneumococcal lineage in this study. Globally, GPSC10 drove the increase in serotypes 19A [51,56] and 24F [57,58] after the introduction of PCV7 and PCV13, respectively. Similar to GPSC1, GPSC10 is also MDR lineage and has high invasive disease potential independent of the serotype [57]. The fourth common pneumococcal lineage GPSC5 was also identified in South Africa, showing an expansion of serotype 35B/D following the introduction of PCV13 [53,59]. The presence of these globally known serotype replacement pneumococcal lineages (GPSC1, 5 and 10), together with the regional lineage (GPSC268) expressing serotype 16F that would not be covered by any childhood PCV soon, underlines the importance of continuous surveillance on pneumococcal carriage and disease populations in Ethiopia during and after the PCV13 introduction.

It is alarming that the prevalence of predicted penicillin resistance was 50–100%, depending on the sample sources, when we applied the CLSI meningitis cutoff. This finding highlighted that if these isolates cause meningitis, penicillin might not be an effective antibiotic option. The penicillin resistance was mainly associated with serotype 19A expressed by GPSC1 and GPSC10. The potential reduction of serotype 19A brought about by PCV13 would potentially reduce penicillin resistance. However, it is of note that GPSC268 (serotype 16F) that is not protected against any upcoming PCVs is also penicillin resistant and occasionally resistant to macrolide (conferred by *mefA*) and tetracycline.

The acquisition of resistance genes is one of the major strategies by which pneumococci and other pathogens respond to the use of antibiotics and ensure the persistence of lineages [46,60]. We also identified resistance determinants that confer resistance to different antibiotics. Concurrently, the most common resistance mechanism identified regarded changes in *folA* and *folP* that confer resistance to sulfamethoxazole-trimethoprim. In Pakistan, 89% of the nasopharyngeal isolates collected 2–6 years after the introduction of PCV10 were resistant to sulfamethoxazole-trimethoprim [61]. Similarly, in Brazil, there was a significant level of resistance to sulfamethoxazole-trimethoprim with changes in *folA/folP*, among *S. pneumoniae* isolates causing IPD, despite a decline in resistance in the post-PCV10 period (38%) as compared with the pre-PCV10 period (57%) [46]. There were no penicillin-resistant *S. pneumoniae* isolates in this study, but intermediate susceptibility to penicillin was seen in 14.6% of the isolates. In Kenya, 2 years after the introduction of PCV10, there was a significant decline in the carriage of PCV10-type penicillin-intermediate *S. pneumoniae* isolates, which made up more than 99% of the penicillin non-susceptible isolates in the pre-PCV10 period [62].

The impact of vaccines on antibiotic resistance is twofold. Not only do they result in a direct reduction of strains that carry antibiotic-resistant genes, since resistance is often higher among strains that are targets of vaccines, but they also reduce the frequency of febrile infections and therefore lead to a reduction in antibiotic use [63].

In the present study, all 15 isolates that were intermediately resistant to penicillin were serotype 19A expressing GPSC1 (ST320) isolates. Serotypes 19A and 16F are among the serotypes that frequently colonize the nasopharynx and among the high-ranking serotypes that have acquired *pbp* fragments from viridans streptococci such as *Streptococcus mitis* [60]. Among all pneumococcal lineages, GPSC10, GPSC59, GPSC9, GPSC5 and GPSC1 are the top five ranking lineages known to acquire *pbp* through horizontal gene transfer [60]. In a study performed in Russia on 179 isolates recovered from cerebrospinal fluid (*n*=77), nasopharyngeal swabs (*n*=99) and other non-sterile site swabs (*n*=3), GPSC1 was among the four most common lineages that accounted for 65% of MDR isolates [47]. In Brazil, results from the genomic characterization of 698 invasive *S. pneumoniae* isolates indicated that the main lineage associated with MDR in the post-PCV10 period was GPSC1 (ST320, serotype 19A) [46]. The expansion of this lineage is thought to have been facilitated by capsular switch events from serotype 19F in the pre-PCV period to 19A in the post- PCV period and association with MDR in the post-PCV period [21,37, 64, 65]. The introduction of PCV13 in the immunization schedule has resulted in sharp and sustained declines in serotype 19A carriage and disease in countries such as the USA, the UK, Norway, Denmark, Israel and South Africa as reviewed by Isturiz *et al.* [22]. However, a recent report from Ireland of vaccine breakthroughs/failure cases due to serotype 19A (ST320) GPSC1 lineage among vaccinated children aged<5 years [23] indicates the importance of continued genomic surveillance even after the introduction of PCV13.

The main limitation of this study is the lack of sequenced isolates from the pre-PCV10 period that would have helped to assess the changes in the population structure before and after PCV10 introduction. A second limitation is the small number of invasive disease isolates and non-uniform representation of carriage and disease isolates. Additionally, we were unable to repeat the Quellung serotyping to confirm the discordance between the results of Quellung and SeroBA.

In conclusion, genomic characterization of paediatric *S. pneumoniae* isolates in Addis Ababa, Ethiopia, showed the distribution of important lineages and antimicrobial resistance determinants 5–6 years after the introduction of PCV10 in Ethiopia. The results indicate that GPSC1 which expresses the non-PCV10, PCV13 unique serotype 19A is predominant in both carriage and disease. This is accompanied by the predominance of MDR ST320. It is therefore important to continue performing genomic surveillance of carriage and disease pneumococcal isolates in Ethiopia to assess the impact of the recently introduced PCV13 on the serotype distribution and population structure of *S. pneumoniae.*

## Supplementary material

10.1099/mgen.0.001376 Uncited Supplementary Material 1.

10.1099/mgen.0.001376 Uncited Supplementary Material 2.

## Abbreviations

AOM: acute otitis media
CC: clonal complex
CLSI: Clinical and Laboratory Standards Institute
DLV: double-locus variant
GPSC: global pneumococcal sequence cluster
IPD: invasive pneumococcal disease
MDR: multidrug resistance
MIC: minimum inhibitory concentration
NVT: non-vaccine type
PBP: penicillin-binding protein
PCV: pneumococcal conjugate vaccine
SLV: single-locus variant
ST: sequence type
VT: vaccine type
WGS: whole-genome sequencing
