# Supplementary material for: Genomic characterization of Streptococcus pneumoniae isolates obtained from carriage and disease among paediatric patients in Addis Ababa, Ethiopia
Source: Microb Genom. 2025 Mar 18;11(3):001376. doi: 10.1099/mgen.0.001376 (PMC11986848; doi:10.1099/mgen.0.001376)
Supplement: Uncited Supplementary Material 1. [file mgen-11-01376-s001.pdf]

Supplementary table 1. Quality control parameters of 119 *Streptococcus pneumoniae* used for selecting sequences for downstream analysis

Lane	*Streptococcus pneumoniae*	Total Length	Number of Contigs	Genome Covered	Depth of Coverage	hetsnp_count	qc
44905_2#114	91.85	2174787	32	92.36	221.6	150	Pass
44905_2#116	92.83	2147978	34	89.21	278.39	65	Pass
44905_2#118	95.09	2056770	30	86.33	194.1	43	Pass
44905_2#120	93.37	2076164	31	88.04	161.04	68	Pass
44905_2#122	93.44	2070786	18	87.97	219.15	77	Pass
44905_2#124	93.07	2099765	24	87.69	177.56	69	Pass
44905_2#126	93.43	2117301	24	89.25	295.96	62	Pass
44905_2#128	94.02	2097298	16	86.82	262.14	63	Pass
44905_2#146	87.37	2130033	25	87.64	228.6	54	Pass
44905_2#148	93.98	2114446	43	86.71	105.06	59	Pass
44905_2#150	94.59	2075989	22	86.4	257.34	55	Pass
44905_2#152	91.03	2182843	56	88.32	178.76	72	Pass
44905_2#154	95.08	2063217	35	86.42	245.16	38	Pass
44905_2#156	92.33	2041360	18	87.56	270.9	37	Pass
44905_2#160	93.17	2162912	36	87.99	225.9	62	Pass
44905_2#178	95.17	2063726	29	86.79	187.48	50	Pass
44905_2#18	94.95	2087358	24	87.79	237.18	80	Pass
44905_2#180	88.42	2152052	32	86.23	83.12	63	Pass
44905_2#182	95.28	2061803	30	86.54	192.2	52	Pass
44905_2#184	94.04	2085515	17	88.08	364.05	54	Pass
44905_2#187	92.68	2116158	27	91.99	123.87	55	Pass
44905_2#189	93.11	2068406	42	89.3	78.82	66	Pass
44905_2#190	93.4	2072366	11	88.9	165.72	57	Pass
44905_2#191	95.09	2039513	30	86.56	103.38	54	Pass
44905_2#192	94.85	2055325	24	86.52	149.04	61	Pass
44905_2#20	80.71	2150846	41	89.95	111.96	80	Pass
44905_2#209	95.05	2056091	33	86.46	48.12	52	Pass
44905_2#210	95.12	2061244	29	86.43	308.7	50	Pass
44905_2#211	92.37	2125173	24	89.76	166.24	67	Pass
44905_2#212	85.3	2150273	42	89.6	79.12	65	Pass
44905_2#214	79.28	2206105	47	89.24	76.74	68	Pass
44905_2#215	74.23	2171007	20	87.8	258.42	48	Pass
44905_2#217	95.24	2053261	23	86.38	88.14	43	Pass
44905_2#22	92.19	2110355	41	86.97	373.95	52	Pass
44905_2#234	80.87	2135660	19	89.14	195.4	57	Pass
44905_2#235	94.86	2028934	22	86.6	101.82	57	Pass
44905_2#236	95.02	2058118	34	86.56	114.9	51	Pass
44905_2#238	92.97	2029089	15	86.57	198.2	51	Pass
44905_2#239	93.61	2098696	27	88.23	128.1	60	Pass

44905_2#24	80.78	2164255	22	88.58	291.12	64	Pass
44905_2#240	95.16	2062778	35	86.44	207.35	51	Pass
44905_2#241	93.89	2111969	31	89.3	295.61	77	Pass
44905_2#258	95	2041327	29	86.42	304.15	52	Pass
44905_2#259	92.69	2089449	13	87.46	78.44	54	Pass
44905_2#260	93.76	2151095	33	90.91	237.48	60	Pass
44905_2#261	93.94	2065531	30	87.94	193.6	45	Pass
44905_2#262	93.79	2059448	26	89.41	73.95	57	Pass
44905_2#264	92.01	2121955	46	86.91	180.8	64	Pass
44905_2#265	93.13	2144517	35	87.68	213.84	54	Pass
44905_2#282	94.23	2158462	30	88.77	161.08	74	Pass
44905_2#283	89.1	2192104	37	89.37	165.6	54	Pass
44905_2#284	92.77	2107259	13	88.92	156.87	52	Pass
44905_2#285	89.99	2156379	27	93.33	292.4	55	Pass
44905_2#286	91.94	2068816	24	88.67	62.59	52	Pass
44905_2#287	93.87	2066282	34	87.33	98.4	59	Pass
44905_2#288	89.82	2111496	24	87.97	127.5	56	Pass
44905_2#289	91.82	2133277	46	86.72	90.14	66	Pass
44905_2#308	89.77	2084912	21	86.8	232.2	60	Pass
44905_2#309	94	2092837	40	86.61	198.35	77	Pass
44905_2#310	85.38	2124528	28	89.99	113.7	65	Pass
44905_2#311	95.05	2103687	29	88	182.2	50	Pass
44905_2#32	95.23	2065300	30	86.42	371.25	50	Pass
44905_2#330	91.16	2163833	39	90.24	72.12	87	Pass
44905_2#331	92.45	2097938	24	88.91	188.32	56	Pass
44905_2#332	94.06	2122413	33	88.47	276.5	55	Pass
44905_2#333	94.41	2165075	36	88.77	233.22	60	Pass
44905_2#334	94.64	2054074	11	86.89	158.85	56	Pass
44905_2#335	84.96	2152142	35	90.58	272.46	95	Pass
44905_2#336	94.21	2086102	28	91.64	346.48	52	Pass
44905_2#337	94.13	2080234	22	91.42	245.15	41	Pass
44905_2#50	91.89	2150996	23	89.25	238.86	70	Pass
44905_2#52	88.72	2157301	39	86.85	137.64	65	Pass
44905_2#54	88.02	2161669	32	89.86	142.71	167	Pass
44905_2#64	91.33	2162919	42	87.8	48.1	86	Pass
44905_2#84	95.08	2058110	31	86.35	251.58	61	Pass
44905_2#86	88.85	2114836	34	87.8	91.14	81	Pass
44905_2#90	91.16	2167782	23	89.97	164.68	73	Pass
44905_2#92	93.86	2022206	17	85.66	285.11	67	Pass
44905_2#94	94.18	2063615	26	86.44	205.3	48	Pass
44905_2#96	93.93	2122801	23	86.75	366.48	72	Pass
45412_2#114	85.57	2199760	35	87.78	76.48	73	Pass
45412_2#116	86.35	2187345	29	90.3	120.33	61	Pass
45412_2#120	90.31	2174745	20	90.15	93.18	71	Pass
45412_2#122	87.46	2197359	44	89.89	67.54	85	Pass
45412_2#124	86.76	2152724	35	87.85	57.39	76	Pass

45412_2#126	85.62	2178729	39	89.84	102.1	79	Pass
45412_2#197	92.53	2094833	13	87.55	120.3	49	Pass
45412_2#198	88.62	2141490	51	86.94	222.84	72	Pass
45412_2#199	94.52	2093416	21	88.57	153	68	Pass
45412_2#200	95.13	2058353	26	86.65	113.26	61	Pass
45412_2#201	93.97	2094147	25	88.48	189.4	61	Pass
45412_2#202	93.21	2127412	27	90.3	100.28	93	Pass
45412_2#203	91.21	2157308	46	87.11	102.18	61	Pass
45412_2#220	93.34	2103394	25	88.35	163.6	66	Pass
45412_2#221	93.36	2085324	32	87.31	64.5	58	Pass
45412_2#223	89.44	2130204	60	87.06	108.64	69	Pass
45412_2#227	93.38	2103001	30	88.56	129.63	65	Pass
45412_2#244	91.54	2153636	53	86.88	114.27	51	Pass
45412_2#245	93.45	2134029	30	89.03	212.15	56	Pass
45412_2#247	92.36	2125050	20	89.06	84.16	72	Pass
45412_2#249	94.86	2056060	29	86.57	92.82	44	Pass
45412_2#251	94.31	2165055	31	88.95	127.14	80	Pass
45412_2#271	95.05	2057767	29	86.67	115.92	45	Pass
45412_2#274	94.9	2055217	27	86.47	80.12	53	Pass
45412_2#275	94.72	2057976	26	86.55	92.26	50	Pass
45412_2#292	94.76	2057603	29	86.46	84	52	Pass
45412_2#294	93.36	2056686	33	87.64	73.34	59	Pass
45412_2#295	91.66	2166896	37	89.06	93.08	65	Pass
45412_2#296	94.12	2055635	27	86.56	95.2	38	Pass
45412_2#297	93.07	2057636	30	86.74	72.89	49	Pass
45412_2#317	88.47	2065992	35	86.61	108.32	63	Pass
45412_2#319	93.71	2091370	34	88.21	90.48	54	Pass
45412_2#32	90.77	2129330	50	87.18	64.84	69	Pass
45412_2#323	87.82	2156400	58	86.98	85.78	63	Pass
45412_2#52	87.97	2154501	59	87.13	57.63	56	Pass
45412_2#54	94.38	2057347	30	86.72	73.87	61	Pass
45412_2#64	90.16	2247263	36	91.28	112.46	82	Pass
45412_2#88	86.96	2158454	41	88.19	103.74	56	Pass
45412_2#90	93	2088858	32	87.14	81.28	60	Pass
44905_2#158	79.88	3213228	1315	87.64	180.25	1168	Fail
44905_2#186	29.69	2101807	193	85.72	73.3	2158	Fail
44905_2#216	94.08	2266048	249	90.86	225.85	1590	Fail
44905_2#237	77.96	2344230	442	91.25	102.9	56	Fail
44905_2#263	91.5	2767246	614	90.08	202.25	2822	Fail
44905_2#28	90.79	2802530	591	93.43	199.3	3492	Fail
44905_2#30	94.97	2153501	58	87.92	262.26	301	Fail
44905_2#306	92.67	2143156	83	90.39	115.02	3623	Fail
44905_2#307	67.26	4042556	46	90.66	187.62	47	Fail
44905_2#312	94.04	2086154	26	92.41	319.06	1989	Fail
44905_2#313	80.64	2518718	370	93.52	231.8	2615	Fail
44905_2#60	48.98	67951	38	89.01	12.24	776	Fail

44905_2#62	22.44	4883637	84	86.68	34.74	123	Fail
44905_2#88	26.59	8499905	2407	91.74	43.53	66	Fail
45412_2#118	91.36	3033600	1643	91.55	116.16	4067	Fail
45412_2#18	86.29	2102577	21	90.93	126.48	782	Fail
45412_2#196	81.17	3983664	56	88.38	175.35	68	Fail
45412_2#20	40.57	49625	13	88.84	18.53	2027	Fail
45412_2#22	2.97	3213946	500	46.15	2.58	18	Fail

Legend: hetsnp, heterozygosity (het) rate for single nucleotide polymorphisms (snp); qc, quality control

Supplementary table 2. *Streptococcus pneumoniae* isolates (n = 26) with discordant Quellung and SeroBA serotyping results

**Pneumococcal isolate names**	**Serotypes by Quellung**	**In silico serotype by SeroBA**	**GPSC**
GPS_ET_LBR_04015	23F	21	5
GPS_ET_LBR_01007B	16F	21	5
GPS_ET_ARI_PD02607E3	1	14	9
GPS_ET_LBR_058	12B	12F	26
GPS_ET_LBR_00149B	NT	19A	53
GPS_ET_LBR_01111B1	18A	18C	61
GPS_ET_LBR_HC00089B	34	9V	61
GPS_ET_LBR_00862B3	15B	15C	88
GPS_ET_LBR_01208B3	15B	15C	88
GPS_ET_LBR_01317B	24F	15B	88
GPS_ET_LBR_00114B1_R1	24A	serogroup 24	91
GPS_ET_LBR_00894B1_R1	19A	2	96
GPS_ET_LBR_01093A_R1	33A	33C	165
GPS_ET_ARI_PD00089B1	34	35B	233
GPS_ET_ARI_PD01364B1	38	35B	233
GPS_ET_LBR_00125B	35A	35C	268

GPS_ET_LBR_04000	33C	17F	411
GPS_ET_LBR_01277B2	24F	serogroup 24	539
GPS_ET_LBR_01321B3	6A	34	584
GPS_ET_ARI_PD01321B3_R1	6A	34	584
GPS_ET_LBR_060	11D	11A	642
GPS_ET_LBR_01117B	35B	11B	645
GPS_ET_LBR_00151B1	9L	9N	699
GPS_ET_LBR_00061B1	33B	33G	867
GPS_ET_LBR_04012	23F	13	1005
GPS_ET_LBR_00830B2	12F	33B	1013

Legend: GPSC, global pneumococcal sequence clusters

Supplementary table 3. GPSCs and serotypes of 103 *Streptococcus pneumoniae* isolates among paediatric patients in Addis Ababa, Ethiopia, isolated between September 2016 and August 2017

GPSC	Associated Serotypes	Number	Isolate sample source	Number
1	19A	15	Blood	1
			MED	6
			NP	8
5	19A	2	MED	1
			NP	1
	23F	1	NP	1
	23A	1	NP	1
	16F	1	NP	1
6	11A	1	MED	1
9	14	1	MED	1

10	19A	8	Blood	1
			MED	3
			NP	4
11	21	1	NP	1
21	19F	1	NP	1
22	15A	1	NP	1
25	15B	1	NP	1
26	12F	2	NP	2
	12B	1	NP	1
32	7F	1	NP	1
43	35A	1	NP	1
	11A	1	NP	1
44	15A	1	NP	1
53	19A	3	NP	3
	NT	1	NP	1
61	18A	1	NP	1
	34	1	NP	1
88	15C	2	NP	2
	15B	1	NP	1
91	24	1	NP	1
95	18A	1	NP	1
96	19A	1	NP	1
116	23A	1	NP	1
165	33C	2	Blood	2
169	23B	1	NP	1
185	6B	1	NP	1
233	35B	1	NP	1

	34	1	NP	1
	38	1	NP	1
268	16F	7	MED	1
			NP	6
	11A	1	NP	1
	35A	1	NP	1
302	35F	2	NP	2
376	6A	3	NP	3
411	33C	1	NP	1
457	11B	1	NP	1
539	24F	1	NP	1
584	34	3	NP	3
	6A	2	NP	2
624	11D	1	NP	1
645	35B	1	NP	1
661	NT	4	NP	4
667	20	1	NP	1
690	6C	1	NP	1
699	9L	1	NP	1
867	33B	1	NP	1
879	19F	2	NP	2
883	21	1	NP	1
883	21	1	NP	1
1004	10A	1	NP	1
1005	13	2	NP	2
1013	33B	1	NP	1
1014	NT	1	NP	1

1015	7C	1	NP	1
1016	NT	1	NP	1

Legend: GPSC, global pneumococcal sequence cluster; MED, middle ear discharge; NP, nasopharyngeal swab

Supplementary table 4. ***Streptococcus pneumoniae*** **isolates with discordant phenotypic and Insilco predicted antimicrobial susceptibility patterns for penicillin and erythromycin**

No	Study Name	Sample Source	Penicillin				Erythromycin	
			Phenotypic meningitis	Phenotypic non-meningitis	Insilico meningitis	Insilico non-meningitis	Phenotypic	Insilico
1	GPS_ET_LBR_01240C3	Blood	R	**S**	R	**I**	R	R
2	GPS_ET_LBR_00864E	MED	R	**S**	R	**I**	R	R
3	GPS_ET_LBR_01101E	MED	R	**S**	R	**I**	R	R
4	GPS_ET_LBR_01078E	MED	R	**S**	R	**I**	R	R
5	GPS_ET_LBR_00878E2	MED	R	**S**	R	**I**	R	R
6	GPS_ET_LBR_02611E2	MED	R	**S**	R	**I**	R	R
7	GPS_ET_LBR_04015	NP	R	S	R	S	**R**	**S**
8	GPS_ET_LBR_01062B	NP	R	**S**	R	**I**	R	R
9	GPS_ET_LBR_00840B2	NP	R	**S**	R	**I**	R	R
10	GPS_ET_LBR_00857B3	NP	**S**	S	**R**	S	**R**	**S**
11	GPS_ET_LBR_04011	NP	R	S	R	S	**R**	**S**
12	GPS_ET_LBR_01603B2	NP	R	S	R	S	**R**	**S**
13	GPS_ET_LBR_00033B2	NP	**S**	S	**R**	S	**R**	**S**
14	GPS_ET_LBR_060	NP	**S**	S	**R**	S	**R**	**S**
15	GPS_ET_LBR_00835B	NP	S	S	S	S	**S**	**R**
16	GPS_ET_LBR_00126B	NP	R	**S**	R	**I**	R	R
17	GPS_ET_LBR_01111B1	NP	S	S	S	S	**R**	**S**
18	GPS_ET_LBR_00072B	NP	R	S	R	S	**R**	**S**
19	GPS_ET_LBR_00946B	NP	R	**S**	R	**I**	R	R
20	GPS_ET_LBR_00076B1	NP	R	**S**	R	**I**	R	R
21	GPS_ET_LBR_00979B	NP	R	**S**	R	**I**	R	R
22	GPS_ET_ARI_PD01364B1	NP	R	S	R	S	**R**	**S**
23	GPS_ET_ARI_PD01228B4	NP	R	S	R	S	**R**	**S**
24	GPS_ET_LBR_00894B1_R	NP	**R**	S	**S**	S	**S**	**R**

Legend: MED, middle ear discharge; NP, nasopharyngeal swab. Those indicated in bold are ones that are discordant.
